# Effects of EECP combined with individualized cardiac exercise rehabilitation on lipoprotein(a) levels in patients with coronary heart disease

**DOI:** 10.1515/med-2026-1398

**Published:** 2026-04-08

**Authors:** Guangyun Cao, Xuemei Zhang, Chunqiao Liu

**Affiliations:** Department of Cardiology, Hebei General Hospital, Shijiazhuang, Hebei, China; Department of Rheumatology and Immunology, Hebei General Hospital, Shijiazhuang, Hebei, China; Department of Medical Care, Hebei General Hospital, Shijiazhuang, Hebei, China

**Keywords:** coronary heart disease, efficacy, enhanced external counterpulsation, individualized cardiac exercise rehabilitation training, lipoprotein(a)

## Abstract

**Objectives:**

This study aimed to evaluate the effects of combining enhanced external counterpulsation (EECP) with individualized cardiac exercise rehabilitation training on lipoprotein(a) levels in patients with coronary heart disease (CHD).

**Methods:**

This retrospective analysis included 122 patients with CHD who received treatment at the hospital between January 2023 and September 2023. 61 patients were assigned to the study group, receiving EECP therapy plus individualized cardiac exercise rehabilitation, while another 61 in the control group were given standard mattress therapy combined with the same exercise rehabilitation. Therapeutic efficacy was evaluated over a 6-month period and compared between groups. In addition, changes in blood lipid parameters were assessed before treatment and 30 days after treatment.

**Results:**

After treatment, no statistically significant changes were observed in total cholesterol, triglycerides, high-density lipoprotein cholesterol, or very-low-density lipoprotein cholesterol (p>0.05). In contrast, low-density lipoprotein cholesterol and lipoprotein(a) levels decreased compared with pretreatment values. During follow-up, the total effective treatment rate in the study group reached 93.83 % at 6 months.

**Conclusions:**

The combination of EECP and individualized cardiac exercise rehabilitation training was associated with a reduction in lipoprotein(a) levels and improved overall cardiovascular status in patients with CHD.

## Introduction

Coronary atherosclerotic heart disease (CHD), also referred to as coronary heart disease, is a cardiovascular condition characterized by atherosclerotic lesions in the coronary arteries that lead to narrowing or obstruction of the vascular lumen. These structural changes reduce coronary blood flow and result in inadequate oxygen and nutrient delivery to myocardial cells, thereby inducing myocardial ischemia and hypoxia. Persistent impairment of myocardial perfusion disrupts normal cellular metabolism and contributes to progressive pathological alterations characteristic of CHD [1].

Epidemiological evidence indicates that the prevalence of CHD varies across occupational groups, with higher rates reported among farmers and industrial workers. The condition occurs more frequently in men than in women and shows a positive association with increasing age [2]. The pathogenesis of CHD is primarily attributed to coronary atherosclerosis, with established risk factors including advanced age, insufficient sleep, smoking, obesity, dyslipidemia, diabetes, and other chronic diseases, as well as lower educational attainment [3].

The predominant clinical manifestations of CHD include angina pectoris and acute coronary syndrome. Without timely and effective intervention, CHD may progress to severe complications, such as heart failure and cardiac arrhythmias, which substantially impair quality of life and adversely impact the prognosis of affected patients [4]. Conventional treatment strategies mainly comprise pharmacological therapy, including antiplatelet agents, lipid-lowering and plaque-stabilizing medications, and coronary vasodilators, as well as invasive procedures when indicated. However, long-term pharmacotherapy may be associated with drug resistance or systemic adverse effects, while invasive interventions carry risks of perioperative and postoperative complications. These limitations may contribute to suboptimal therapeutic outcomes or reduced treatment adherence, underscoring the need for more effective and acceptable therapeutic approaches [5].

In recent years, cardiac rehabilitation has been recognized as an essential component of comprehensive cardiovascular management. Enhanced external counterpulsation (EECP) is a non-pharmacological intervention that has been widely applied in clinical practice and has demonstrated effectiveness in alleviating myocardial ischemic symptoms, improving exercise tolerance, and enhancing quality of life in patients with cardiovascular diseases [6], 7]. EECP increases coronary perfusion and optimizes myocardial oxygen supply through sequential external compression of the lower limbs during diastole. In addition, this intervention reduces myocardial oxygen consumption and improves cardiac function, with clinically relevant benefits observed in patients with cardiac insufficiency.

Evidence indicates that EECP may delay the progression of coronary atherosclerosis by improving myocardial perfusion, enhancing vascular endothelial function, and attenuating inflammatory responses [8], 9]. Atherosclerosis is closely associated with dyslipidemia, particularly elevated levels of low-density lipoprotein cholesterol (LDL-C) and lipoprotein(a), which contribute to plaque formation and thrombotic processes. From a mechanistic perspective, improvements in the vascular microenvironment induced by EECP may influence lipid metabolism. However, whether EECP directly or indirectly regulates key lipid parameters, such as lipoprotein(a) and LDL-C, and the extent and specificity of such effects remain unclear.

Individualized cardiac exercise rehabilitation training has been demonstrated to improve lipid metabolism, yet clinical evidence is limited regarding whether its combination with EECP produces additional lipid-regulating benefits through synergistic mechanisms. Therefore, the present investigation aimed to evaluate the clinical efficacy of EECP in patients with CHD, with particular emphasis on the effects of EECP combined with individualized cardiac exercise rehabilitation training on lipid profiles, especially lipoprotein(a), thereby providing evidence supporting more precise therapeutic strategies for CHD.

## Materials and methods

### General information

This retrospective analysis included 122 patients diagnosed with CHD who received treatment at the hospital between January 2023 and September 2023. All patients met the diagnostic criteria specified in the Chinese expert consensus on CT examination and diagnosis of coronary heart disease and the primary care guidelines for stable coronary artery disease (2020) [10], 11]. Patients were assigned to either a study group (n=61) or a control group (n=61). Patients in the study group received EECP therapy combined with individualized cardiac exercise rehabilitation in addition to standard pharmacological treatment, whereas those in the control group received standard therapy consisting of mattress therapy combined with individualized cardiac exercise rehabilitation. No statistically significant differences were observed in baseline demographic or clinical characteristics between the two groups (p>0.05) (Table 1). Ethical approval for this investigation was obtained from the Ethics Committee of the hospital (No. 74 of the 2021 Research Ethics Review).

**Table 1:** Baseline clinical characteristics of patients with CHD in the two groups.

Items	Study group (n=61)	Control group (n=61)
Male (example %)	35 (57.4)	37 (60.1)
Age, years	58.976 ± 10.187	61.058 ± 11.600
Smoking history (examples %)	38 (62.3)	37 (60.1)
History of hypertension (cases %)	34 (55.7)	35 (57.4)
Diabetes (cases %)	18 (29.5)	19 (31.1)
Total cholesterol	4.45 ± 0.41	4.34 ± 0.42
Triglyceride	1.51 ± 0.24	1.49 ± 0.25
High-density lipoprotein cholesterol	1.23 ± 0.28	1.21 ± 0.29
Low-density lipoprotein cholesterol	2.19 ± 0.35	2.21 ± 0.33
Very low-density lipoprotein cholesterol	2.39 ± 0.42	2.40 ± 0.41
Lipoprotein(a)	229.8 ± 231.62	230.6 ± 230.71
EDD, mm	47.718 ± 4.769	48.625 ± 4.600
LVEF, %	56.075 ± 7.851	55.9196 ± 8.436
BNP, pg/mL	877.028 ± 778.882	864.289 ± 713.365
Creatinine, μmol/L	74.737 ± 17.084	75.725 ± 16.174
Aspirin	61 (100)	61 (100)
Nitrate esters	41 (33.1)	38 (31.7)
ARB/ACEI	83 (66.9)	78 (65.0)
Beta-blockers	109 (87.9)	101 (84.2)
Calcium ion antagonists	3 (2.4)	4 (3.3)
Statins	116 (93.5)	114 (95.0)
Proton pump inhibitor	61 (49.2)	57 (47.5)
SAQ quality of life, mean (SD)		
The degree of physical activity limitation	80.2 (16.8)	80.5 (17.2)
Stability of angina pectoris	61.0 (22.8)	61.5 (22.8)
Frequency of angina pectoris	81.3 (20.1)	82.1 (19.5)
Treatment satisfaction	74.1 (15.0)	73.6 (14.9)
Disease cognition	59.2 (20.8)	59.7 (20.0)

There was no statistically significant difference in the comparison of general data (p>0.05).

### Inclusion and exclusion criteria

#### Inclusion criteria

Participants were eligible for inclusion if they met all of the following criteria:

(1) Met the diagnostic criteria for chronic stable CHD;
(2) Aged ≥18 years;
(3) Presence of myocardial ischemia-related symptoms, with clinical stability maintained for at least one month;
(4) History of acute myocardial infarction occurring more than one year prior to enrollment;
(5) History of percutaneous coronary intervention (PCI) or coronary artery bypass grafting (CABG) performed more than one year prior;
(6) Evidence of ≥50 % stenosis in at least one major coronary artery branch, confirmed by coronary computed tomography angiography or coronary angiography;
(7) Voluntary participation with provision of written informed consent;
(8) Demonstrated good adherence to treatment.

#### Exclusion criteria

Participants meeting any of the following criteria were excluded:

(1) Myocardial infarction or coronary revascularization (CABG or PCI) within the past 12 months;
(2) Planned CABG or PCI during the study period;
(3) Severe cardiovascular conditions, including persistent severe angina pectoris (Canadian Cardiovascular Society class IV), refractory heart failure, cardiogenic shock, severe aortic valve stenosis or insufficiency, or severe respiratory diseases;
(4) Poorly controlled diabetes mellitus;
(5) Poorly controlled hypertension, defined as persistent elevated blood pressure at the end of screening despite antihypertensive therapy, or systolic blood pressure ≥180 mmHg or diastolic blood pressure ≥110 mmHg;
(6) Severe hepatic or renal disease, including active liver disease, liver cirrhosis, or uremia;
(7) Other serious conditions, such as malignant tumors, severe anemia, or severe renal artery stenosis;
(8) Inability or unwillingness to provide informed consent;
(9) Pregnancy or plans for conception;
(10) Discontinuation of treatment due to personal reasons;
(11) Determined by clinical investigators to be unsuitable for participation.

### Methods

Both groups received standard pharmacological therapy, including aspirin enteric-coated tablets at a dose of 0.1 g once daily and atorvastatin at a dose of 20 mg once daily, along with other indicated medications. In addition to standard drug therapy, patients in the study group underwent EECP therapy combined with individualized cardiac exercise rehabilitation, whereas those in the control group received mattress therapy combined with individualized cardiac exercise rehabilitation.

#### EECP treatment procedure

The EECP treatment was performed according to a standardized protocol, as detailed below:

(1) Equipment: EECP therapy was administered using an EECP device approved by the National Medical Products Administration (Approval No. 20243090711, model OM-A-ECP1), manufactured by Omay (Guangzhou) Med Technologies Co., Ltd.
(2) Inspection and setup: After device activation, the pneumatic cuffs were fully spread to expel residual air, and the system was inspected to confirm the absence of air leakage.
(3) Electrode installation: Electrocardiogram electrode pads were affixed to areas minimally affected by pneumatic cuff vibration, ensuring an upward R-wave configuration. Lead wires were securely connected and fixed in a U-shaped arrangement using adhesive tape.
(4) Patient preparation: Patients were positioned in the supine position and fitted with a finger probe, with alignment ensuring that the sacral region corresponded to the center of the gluteal cuff.
(5) Cuff installation: Pneumatic cuffs were sequentially applied to the calves, thighs, and buttocks.
(6) Pressure adjustment: Initial inflation pressure was set at 40 mmHg above the upper limb systolic blood pressure and gradually increased to the maximum level tolerated by the patient, taking age and body habitus into consideration.
(7) Treatment monitoring: The ratio of the diastolic augmentation pressure wave to the systolic pressure wave was continuously monitored. If the ratio decreased below 1.2, inflation and deflation timing were adjusted and the system was re-evaluated for potential air leakage.
(8) Treatment adjustment: Pressure retention time was adjusted based on patient tolerance and heart rate to ensure individualized treatment delivery.
(9) Continuous treatment: EECP therapy was continued until completion of the prescribed treatment course, with ongoing parameter optimization for each patient.

#### Preparation before EECP treatment

To ensure treatment safety and effectiveness, the following preparatory measures were implemented:

(1) Patients were instructed to empty the bladder and limit fluid intake before treatment.
(2) Treatment was avoided during fasting or immediately after excessive food intake.
(3) Consumption of tea, alcohol, and tobacco was prohibited before treatment.
(4) Patients were required to wear close-fitting elastic cotton trousers to facilitate treatment.

#### Monitoring during EECP treatment

During EECP therapy, continuous monitoring was performed as follows:

(1) Patients were maintained in a quiet and relaxed environment.
(2) Vital signs, including blood pressure and oxygen saturation, were continuously monitored.
(3) The diastolic augmentation to systolic pressure ratio was observed and maintained within an appropriate range.
(4) Treatment parameters were adjusted in real-time based on monitoring results.
(5) Treatment was immediately discontinued if abnormal clinical conditions were observed.

#### Post-treatment nursing care

After completion of EECP therapy, the following care measures were applied:

(1) Pneumatic cuffs were gradually deflated in the correct sequence.
(2) Electrocardiogram lead wires and electrode pads were removed.
(3) Patients were asked to report any discomfort.
(4) Patients were encouraged to drink a small amount of warm boiled water.
(5) Patients were observed at rest for 5 min and were permitted to leave the treatment area only after confirmation of absence of discomfort or adverse symptoms.

#### Air mattress therapy

For mattress therapy, patients lay in the supine position on an air mattress that intermittently inflated and deflated. All remaining procedures, monitoring processes, and post-treatment care were identical to those applied during EECP therapy.

#### Individualized cardiac exercise rehabilitation training program

The individualized cardiac exercise rehabilitation training program included the following components:

(1) Types of exercise: Aerobic exercise, beginning with slow walking for 10–15 min per session and gradually increasing to 30 min, along with respiratory training such as deep breathing and lung capacity exercises.
(2) Exercise frequency: Sessions were conducted 3 to 5 times per week and adjusted according to patient tolerance.
(3) Intensity monitoring: Exercise intensity was monitored to maintain heart rate within 50–70 % of the maximum predicted heart rate.
(4) Safety measures: All exercise sessions were conducted under medical supervision, with healthcare personnel prepared to manage emergencies.
(5) Home exercise program: A simplified exercise program was provided upon discharge.
(6) Nutrition and lifestyle guidance: Individualized dietary recommendations and lifestyle guidance were provided.
(7) Psychological support: Psychological counseling and stress management interventions were offered.
(8) Regular assessment: Comprehensive rehabilitation assessments were conducted every 4–6 weeks, with adjustments made as needed.

### Observation indicators

The following parameters were monitored to assess the clinical effects of the intervention:

(1) Blood lipid parameters: Total cholesterol; triglycerides; high-density lipoprotein cholesterol (HDL-C); LDL-C; very-low-density lipoprotein cholesterol (VLDL-C); and lipoprotein(a).
(2) Muscle enzyme indicators: Creatine phosphokinase (CPK); creatine phosphokinase isoenzyme MB (CK-MB); serum lactate dehydrogenase (LDH); aspartate aminotransferase (AST); and alanine aminotransferase (ALT).
(3) Therapeutic efficacy evaluation: Treatment effectiveness was assessed at the 6-month follow-up and categorized as follows [12]:

Significant efficacy: Myocardial indicators and lipid parameters were within the normal range, chest pain symptoms completely resolved, and patients were able to perform tasks requiring physical labor.

Effective: Myocardial indicators and lipid parameters demonstrated improvement, chest pain symptoms were reduced, and strenuous activity could induce symptoms.

Ineffective: Myocardial indicators and lipid parameters remained abnormal, chest pain symptoms did not improve, and patients were unable to tolerate even simple activities.

The total effective treatment rate was calculated using the following formula:

Total effective rate of treatment = (number of cases with significant efficacy + number of effective cases)/total number of patients participating in the study × 100 %.

### Statistical analysis

Sample size calculation was performed using PASS 2021 software, and statistical analyses were conducted using SPSS version 23.0.

Based on previous literature, the mean change in triglycerides before and after treatment was 0.54 in the study group and 0.15 in the control group, with a pooled standard deviation of 0.70 [13]. With a two-sided α=0.05 and statistical power (1−β)=0.80, the minimum required sample size was calculated as 52 cases per group. Considering a 15 % dropout rate, the adjusted sample size was 60 cases per group. Therefore, a total of 120 patients were required for inclusion.

Continuous variables, including lipid profiles and enzymatic indicators, were assessed for distribution using the Shapiro–Wilk test. Data conforming to a normal distribution were presented as mean ± standard deviation, whereas non-normally distributed data were presented as median (interquartile range). For normally distributed continuous variables, the independent-samples *t* test was applied, whereas the Mann Whitney *U* test was used for non-normally distributed variables. Categorical variables, including treatment efficacy and sex distribution, were presented as n (%) and were compared using the chi-squared test; Fisher’s exact test was applied when expected frequencies were <5. For within-group comparisons, paired-samples *t*-tests were used for normally distributed variables, and the Wilcoxon signed-rank test was applied for non-normally distributed variables. A p value <0.05 was considered statistically significant.

### Clinical trial number

Not applicable.

### Ethics approval and consent to participate

This study was conducted with approval from the Ethics Committee of Hebei General Hospital (No. 74 of the 2021 Research Ethics Review). This study was conducted in accordance with the declaration of Helsinki. Written informed consent was obtained from all participants.

## Results

### Comparison of blood lipid parameters

No statistically significant differences were observed in total cholesterol, triglycerides, high-density lipoprotein cholesterol, or very-low-density lipoprotein cholesterol between the control group and the study group before or after treatment (p>0.05). After treatment, low-density lipoprotein cholesterol levels were significantly lower in the study group than in the control group (1.82 ± 0.36 vs. 2.18 ± 0.45 mmol/L; p<0.001) (Table 2). In addition, post-treatment lipoprotein(a) levels were significantly lower in the study group compared with the control group (194 ± 138.19 vs. 235.5 ± 232.70 mg/L; p<0.001) (Table 3).

**Table 2:** Comparison of post-treatment blood lipid parameters between the study group and the control group (±s, mmol/L).

Group	TC	TG	HDL	LDL	VLDL
Control group (n=61)	4.83 ± 0.37	1.46 ± 0.23	1.20 ± 0.20	2.18 ± 0.45	2.41 ± 0.50
Study group (n=61)	4.67 ± 0.32	1.25 ± 0.15	1.19 ± 0.31	1.82 ± 0.36	2.34 ± 0.32
*T*	7.547	10.161	4.635	8.121	8.642
p	0.457	0.342	0.251	<0.001	0.643

p<0.05, statistically significant.

**Table 3:** Comparison of post-treatment Lp(a) levels between the study group and the control group (±s, mg/L).

Group	Lp(a), mg/L
Control group (n=61)	235.5 ± 232.70
Study group (n=61)	194 ± 138.19^a^
*T*	4.612
p	<0.001

^a^Compared with the control group, p<0.05, statistically significant.

### Comparison of therapeutic efficacy

At the post-treatment evaluation, the total effective rate in the study group was 93.83 %, including 30 cases classified as having significant efficacy and 46 cases classified as effective, indicating superior therapeutic outcomes.

### Adverse events

No adverse events were reported during the treatment period.

## Discussion

As the global population ages, the prevalence of cardiovascular diseases, particularly CHD, has increased markedly. CHD is associated with a high disease burden and, in the absence of timely and effective intervention, may lead to life-threatening outcomes for affected patients. Accordingly, the implementation of appropriate and effective management strategies for CHD is of critical importance [14], 15]. Conventional therapeutic approaches primarily include pharmacological therapy and surgical interventions. However, prolonged pharmacological treatment may be accompanied by adverse effects, which can negatively affect treatment adherence. Surgical interventions can improve myocardial ischemia and cardiac function, but their invasive nature and the potential for procedure-related complications continue to be significant limitations in clinical practice [14].

EECP is a non-invasive physical therapy that augments arterial blood flow toward the aorta during diastole through the cyclical inflation and deflation of pneumatic cuffs applied to the calves, thighs, and buttocks. This mechanism increases coronary perfusion and supports adequate myocardial oxygen supply. EECP has been demonstrated to improve cardiac function and reduce cardiac afterload [6]. Due to the absence of invasive or traumatic procedures, EECP is associated with a favorable safety profile and high treatment adherence. Its non-invasive characteristics make it particularly suitable for patients with CHD, especially those who are unable to tolerate surgical interventions or intensive pharmacological therapy. Clinical evidence indicates that EECP is associated with reductions in angina symptoms and improvements in exercise tolerance and quality of life, along with a decreased incidence of cardiovascular complications [6], 16], 17]. As an adjunctive therapeutic modality, EECP represents a viable treatment option for patients with CHD.

As a form of non-invasive circulatory support therapy, EECP influences pathological processes associated with coronary heart disease through multiple mechanisms. By increasing cardiac perfusion, EECP augments shear stress on vascular walls, which contributes to reduced endothelial injury and improved endothelial function. This hemodynamic effect is accompanied by attenuation of inflammation related to endothelial dysfunction, thereby inhibiting the progression of coronary atherosclerosis. Collectively, these changes support adequate myocardial oxygenation and perfusion, reduce left ventricular energy expenditure, and promote overall disease recovery [18]. In addition, previous evidence indicates that EECP is associated with reductions in inflammatory markers, including procalcitonin, in patients with CHD, highlighting its potential role in modulating systemic inflammatory status and improving routine blood indicators [19].

HDL-C is recognized as a protective factor in cardiovascular health, whereas elevated levels of triglycerides, LDL-C, and VLDL-C are established risk factors for cardiovascular injury. Increased concentrations of triglycerides, LDL-C, and VLDL-C promote lipid deposition within the vascular wall, impair blood flow, and aggravate ischemic conditions by limiting oxygen delivery to tissues [20]. Reduction of these lipid parameters decreases lipid accumulation and supports vascular integrity, thereby facilitating cardiovascular recovery. Increases in HDL-C confer additional protective effects by inhibiting oxidative modification of LDL-C, reducing monocyte adhesion, and attenuating inflammatory processes within the vascular endothelium, which collectively slow disease progression. Abnormal lipid profiles commonly observed in patients with CHD are also closely associated with systemic inflammation, further exacerbating endothelial injury and promoting atherosclerotic development.

EECP may contribute to these beneficial effects through multiple physiological mechanisms. The therapy is associated with reductions in platelet aggregation and vascular endothelial cell proliferation, thereby limiting endothelial injury. Increased vascular shear stress generated during EECP supports endothelial repair, suppresses atherosclerotic plaque formation, and promotes microvascular regeneration. In addition, the enhancement of blood flow to myocardial tissue during EECP may facilitate lipid transport and clearance, thereby reducing lipid accumulation and supporting cardiovascular recovery [21].

A notable finding of the present analysis was the significant reduction in lipoprotein(a) levels after treatment compared with pretreatment values, indicating a potential association between EECP-based intervention and modulation of this cardiovascular risk factor. Lipoprotein(a) is a lipoprotein particle that is strongly associated with increased cardiovascular risk and is composed of a lipid core, apolipoprotein B-100, and the distinctive apolipoprotein(a) [Apo(a)]. Apolipoprotein(a) shares structural homology with plasminogen, although its precise physiological function remains incompletely defined. Circulating lipoprotein(a) levels are largely determined by genetic factors, and elevated concentrations are linked to cardiovascular disease through mechanisms involving atherosclerosis, thrombosis, inflammation, and oxidative stress [22]. The observed reduction in lipoprotein(a) levels following EECP treatment may be related to improvements in myocardial ischemic symptoms, exercise tolerance, and vascular endothelial function. By increasing shear stress on the vascular wall and supporting endothelial repair, EECP may indirectly influence pathways associated with lipoprotein(a)-related vascular injury and attenuate its adverse cardiovascular effects [23].

Based on current medical knowledge, the mechanisms through which enhanced external counterpulsation combined with exercise rehabilitation may influence lipoprotein(a) remain largely inferential, as high-level clinical evidence confirming a consistent or direct reduction in lipoprotein(a) levels with this combined intervention is lacking. Lipoprotein(a) is synthesized primarily in the liver and is predominantly determined by genetic factors, rendering it relatively resistant to modification through dietary measures or conventional lipid-lowering therapies. EECP may indirectly mitigate the pathogenic effects of lipoprotein(a) by improving endothelial function via increased shear stress, enhancing nitric oxide bioavailability, and reducing systemic inflammation and oxidative stress, although its effect on hepatic lipoprotein(a) synthesis remains unclear. Exercise rehabilitation similarly exerts anti-inflammatory and antioxidant effects and improves overall lipid metabolism, but its impact on circulating lipoprotein(a) concentrations has been inconsistent. When applied in combination, EECP and exercise rehabilitation may provide complementary benefits by enhancing endothelial integrity, suppressing inflammatory responses, and improving overall cardiovascular and metabolic homeostasis. These systemic effects may attenuate the atherogenic influence of lipoprotein(a), even if circulating levels do not undergo substantial change.

At present, conventional lipid-lowering therapies, including statins and proprotein convertase subtilisin/kexin type 9 (PCSK9) inhibitors, demonstrate limited efficacy in regulating lipoprotein(a) levels. Although PCSK9 inhibitors have been demonstrated to produce modest reductions in lipoprotein(a), these effects are generally insufficient to support routine clinical use for this specific indication, and approval by the United States Food and Drug Administration for the explicit purpose of lipoprotein(a) reduction has not been granted [22]. This limitation highlights the need for alternative therapeutic strategies specifically targeting lipoprotein(a).

Emerging nucleic acid–based therapies, including antisense oligonucleotides and small interfering RNA agents, have demonstrated potential for reducing lipoprotein(a) levels. For instance, lepodisiran, a siRNA-based therapy, has demonstrated efficacy in Phase I clinical trials, in which a single subcutaneous administration was associated with a sustained reduction in lipoprotein(a) levels for up to 48 weeks and an acceptable safety profile [24]. The integration of these novel therapeutic approaches with the vascular benefits associated with enhanced external counterpulsation may represent a complementary strategy for mitigating lipoprotein(a)-related cardiovascular risk and improving management options for patients with coronary heart disease and elevated lipoprotein(a) levels.

In addition to pharmacological interventions, lifestyle modification has been proposed as a potential approach to influencing lipoprotein(a) levels, although the available evidence remains limited. Adoption of healthy lifestyle behaviors, including increased physical activity and adherence to a balanced diet, has been demonstrated to reduce overall cardiovascular disease risk; however, the direct impact of such interventions on circulating lipoprotein(a) levels requires further clarification [22].

Cardiac rehabilitation programs that integrate EECP with individualized cardiac exercise rehabilitation training may provide a more comprehensive strategy for improving clinical outcomes. This combined approach has been associated with improvements in exercise tolerance, sleep quality, and psychological well-being, including reductions in anxiety and depressive symptoms. Although the specific mechanisms through which this regimen influences lipoprotein(a) levels remain unclear, the overall enhancement of cardiovascular health and vascular function may indirectly mitigate lipoprotein(a)-associated cardiovascular risk [25].

EECP therapy increases arterial and collateral blood flow, thereby improving myocardial oxygenation and perfusion. The intervention promotes the release of endothelial growth factors, supports endothelial repair, and induces neovascularization, which contributes to the development of collateral circulation. These effects reduce myocardial injury and alleviate clinical symptoms such as dyspnea and angina, leading to improvements in functional capacity and quality of life [21]. Reductions in low-density lipoprotein cholesterol have been demonstrated to relieve myocardial ischemic symptoms and improve myocardial function [26]. However, no significant changes in low-density lipoprotein cholesterol levels were observed before and after treatment in the present analysis, indicating that further large-sample clinical investigations are required.

As demonstrated in this study, the addition of individualized cardiac exercise rehabilitation training to EECP was associated with enhanced therapeutic benefits. Individualized exercise rehabilitation is designed to improve cardiopulmonary function, physical capacity, and overall quality of life through tailored training programs [27]. In the study group, 76 patients demonstrated clinical improvement, corresponding to a total effective rate of 93.83 %. These results support a synergistic effect of EECP combined with individualized cardiac exercise rehabilitation training, underscoring their combined value in improving cardiac function and alleviating physical symptoms in patients with cardiac insufficiency.

The observed benefits of this combined intervention may be attributed to complementary mechanisms of action. Both enhanced external counterpulsation and individualized cardiac exercise rehabilitation offer advantages in improving blood lipid parameters, myocardial enzyme indices, and routine blood indicators, thereby contributing to improved clinical outcomes in patients with coronary heart disease or cardiac insufficiency. As a non-invasive and well-tolerated therapy, EECP is particularly suitable for long-term application and represents a feasible alternative for patients who are unable to tolerate invasive procedures or intensive pharmacological regimens. Future investigations should further examine the long-term efficacy of EECP combined with individualized cardiac exercise rehabilitation and evaluate its effects across diverse patient populations, particularly among older individuals and those with multiple comorbid conditions.

Despite the observed effectiveness of EECP combined with individualized cardiac exercise rehabilitation training in improving cardiac function and alleviating physical symptoms in patients with cardiac insufficiency, several limitations should be acknowledged. First, the relatively short follow-up duration limited the assessment of sustained efficacy and long-term safety. Second, the absence of external validation may restrict the generalizability of the findings to broader clinical settings. Third, the modest sample size reduced representativeness with respect to demographic diversity, including age, sex, and comorbidity profiles. Future studies should therefore incorporate larger and more heterogeneous populations with extended follow-up periods to enable a more comprehensive evaluation of long-term outcomes and safety. Such efforts may also facilitate the development of more tailored strategies to enhance the clinical applicability of EECP and individualized cardiac rehabilitation.

## Conclusions

The findings of this study indicate that the combination of enhanced external counterpulsation and individualized cardiac exercise rehabilitation training is a safe and effective therapeutic approach for patients with coronary heart disease. This combined intervention was associated with a reduction in lipoprotein(a) levels, an established cardiovascular risk factor, as well as favorable overall therapeutic efficacy. Although improvements were observed primarily in selected lipid parameters and clinical outcomes, the results support the potential clinical value of integrating EECP with individualized exercise rehabilitation in the management of coronary heart disease. Future investigations should focus on optimizing combined treatment protocols and evaluating their effectiveness across broader clinical contexts, including patients with different disease severities, comorbidity profiles, and demographic characteristics, to further clarify long-term benefits and enhance clinical applicability.
